# Identification of a 3-Gene Model as Prognostic Biomarker in Patients With Gastric Cancer

**DOI:** 10.3389/fonc.2022.930586

**Published:** 2022-07-14

**Authors:** Siming Xue, Tianjiao Zheng, Juan Yan, Jinmin Ma, Cong Lin, Shichen Dong, Chen Wei, Tong Li, Xiaoyin Zhang, Guibo Li

**Affiliations:** ^1^ Beijing Genomics Institute (BGI)-Shenzhen, Shenzhen, China; ^2^ Beijing Genomics Institute (BGI)-Henan, BGI-Shenzhen, Xinxiang, China; ^3^ Beijing Genomics Institute (BGl) College & Henan Institute of Medical and Pharmaceutical Sciences, Zhengzhou University, Zhengzhou, China; ^4^ Department of Gastroenterology, National Clinical Research Center of Infectious Disease, The Third People’s Hospital of Shenzhen, The Second Affiliated Hospital of Southern University of Science and Technology, Shenzhen, China

**Keywords:** gastric cancer, RNA-seq, cox regression, immune microenvironment, survival

## Abstract

**Objective:**

Although the incidence of gastric cancer (GC) is decreasing, GC remains one of the leading cancers in the world. Surgical resection, radiotherapy, chemotherapy, and neoadjuvant therapy have advanced, but patients still face the risk of recurrence and poor prognosis. This study provides new insights for assessment of prognosis and postoperative recurrence of GC patients.

**Methods:**

We collected paired cancer and adjacent tissues of 17 patients with early primary GC for bulk transcriptome sequencing. By comparing the transcriptome information of cancer and adjacent cancer, 321 differentially expressed genes (DEGs) were identified. These DEGs were further screened and analyzed with the GC cohort of TCGA to establish a 3-gene prognostic model (*PLCL1*, *PLOD2* and *ABCA6*). At the same time, the predictive ability of this risk model is validated in multiple public data sets. Besides, the differences in immune cells proportion between the high- and low-risk groups were analyzed by the CIBERSORT algorithm with the Leukocyte signature matrix (LM22) gene signature to reveal the role of the immune microenvironment in the occurrence and development of GC.

**Results:**

The model could divide GC samples from TCGA cohorts into two groups with significant differences in overall and disease-free survival. The excellent predictive ability of this model was also validated in multiple other public data sets. The proportion of these immune cells such as resting mast cells, T cells CD4+ memory activated and Macrophages M2 are significantly different between high and low risk group.

**Conclusion:**

These three genes used to build the models were validated as biomarkers for predicting tumor recurrence and survival. They may have potential significance for the treatment and diagnosis of patients in the future, and may also promote the development of targeted drugs.

## Introduction

Gastric cancer is one of the leading causes of cancer deaths worldwide. There are many factors in the pathogenesis of GC, such as genetic factors, age, sex, eating habits, viral infections, and Helicobacter pylori infection (1). Great efforts have been made to improve the prognosis for GC patients. Surgery, radiotherapy, neoadjuvant chemotherapy, and molecular targeted therapy are commonly used therapies for GC (2). Despite advances in treatment, the survival rate is low, and the prognosis of patients remains poor due to the heterogeneity of tumors. Therefore, understanding the complicated pathogenesis of GC and the related factors affecting its survival or recurrence is very important.

Nowadays, sequencing technology has evolved to reveal changes in disease evolution at the genetic level (3). However, the tissue phenotypes of normal and diseased states of patients are different, so it is necessary to attempt a fully understanding of the genes and biological pathways that are active in different states (4). Early stage is the best time to study the pathogenesis of gastric cancer, and its physiological and immunological changes may also be involved in the progression of advanced stage tumors. Therefore, key candidate genes related to the occurrence, development and prognosis of diseases can be obtained through comparison with normal tissues, which will help to screen specific biomarkers to provide new ideas for disease prevention and personalize treatment of patients.

In the current study, in order to evaluate the key genes related to the prognosis of early GC, we analyzed the RNA-seq data from 17 early GC patients, each of whom provided paired tumor-adjacent area, and then combined the data with public databases for comprehensive analysis. We performed gene function enrichment analysis on DEGs between the tumor and the adjacent area. After which, we screened out genes related to survival from DEGs by combining GC samples and clinical information from different regions and races in The Cancer Genome Atlas (TCGA) and established a 3-gene model through Lasso and multi-factor Cox regressions. The survival risk model was subsequently verified in three independent GEO data sets. In addition, the differences in immune cells proportion between the high- and low-risk groups were analyzed to reveal the role of the immune microenvironment in the occurrence and development of GC. This study promoted the identification of specific genes as potential biomarkers to predict tumor recurrence and survival time based on the analysis of differential genes, and explained the possible reasons for differences in survival by analyzing immune cells proportion. Our study further deepens the understanding of the molecular basis of gene regulation, and may provide valuable insight on evaluating the potential molecular mechanisms of cancer, detecting disease markers and developing new targeted anticancer drugs.

## Materials and Methods

### Tissue Samples and Processing

We collected cancer and adjacent tissues of 17 patients **(** **Supplementary Tables 1, 2** **)** with early primary GC who underwent gastrectomy in the Third People’s Hospital of Shenzhen in 2020. Written informed consent was obtained from these patients, and has been approved by the Institutional Review Board of BGI (BGI-IRB 20170-T1). All patients with *Helicobacter pylori* positive did not receive antitumor therapy before surgery. Tissues were stored in liquid nitrogen at -196°C once obtained from the surgery. The samples were sent for RNA sequencing. The sequencing company is BGI-Shenzhen Co., Ltd. The sequencing platform is DNBSEQ, paired-end sequencing.

### Analyses of Differentially Expressed Genes and Enrichment Analysis

In this study, RSEM software was used for gene expression quantification (5). We determined the DEGs between normal tissues and tumor tissues through DEseq2 (6). The screening threshold is |logFC|>1, Padj < 0.05. The R package ‘ClusterProfile’ was used to process the Gene Ontology (GO) enrichment analysis for the DEGs.

We used the “c2: KEGG subset of CP” from MSigDB downloaded from http://www.gsea-msigdb.org/gsea/login.jsp as a gene reference set and conducted Gene Set Enrichment Analyseis for the ranked all genes from tumor and adjacent by Log2FoldChange (7).

### Construction and Verification of Prognosis Model

The R package ‘TCGAbiolinks’ was used to download 347-row RNA-Seq information of GC with matched clinical information from the TCGA database, with cancer type=“TCGA-STAD”. After further processing of 347 GC transcriptome data, the 321 DEGs were identified and the corresponding expression matrix (FPKM) were extracted.

The best separation method from R package ‘survminer’ was used to screen (minprop=0.45, *P*<0.05) survival-related genes then LASSO (Least absolute shrinkage and selection operator) regression was performed to select more representative genes. Finally, multi-dimensional Cox regression was performed on the genes obtained in the previous step to construct a hazard ratio model (8).

We used the R package ‘SurvivalROC’ to draw a 3-5 years ROC curve and evaluate the predictive ability of the model by the AUC value. TCGA-STAD data and GEO data sets (GSE15459, GSE84437, GSE62254) were used to evaluate model expansibility. Univariate and multivariate Cox regression survival models were used to evaluate the clinical characteristics and risk scores of GC patients.

### Relation Between Immune Infiltration and Risk Score

We discriminate 22 human immune cell phenotypes by the CIBERSORT algorithm with the Leukocyte signature matrix (LM22) gene signature (9). We used a T-test to evaluate the differences between different risk groups (*P*<0.05) and used Pearson correlation analysis to assess the correlation between risk score and immune cells (*P*<0.05).

### Equations

The equations of risk score obtained from the training set:


(1) riskscore=0.22686×PLCL1+0.03648×PL0D2+0.23693×ABCA6

### Statistical Analysis

All statistical analyses were performed using R. Two-sided paired or unpaired Student’s t-tests and the unpaired Wilcoxon rank-sum test were used where indicated. *P*< 0.05 was considered statistically significant.

## Results

### Acquisition of Differential Genes and Enrichment Analysis

**Figure 1:**
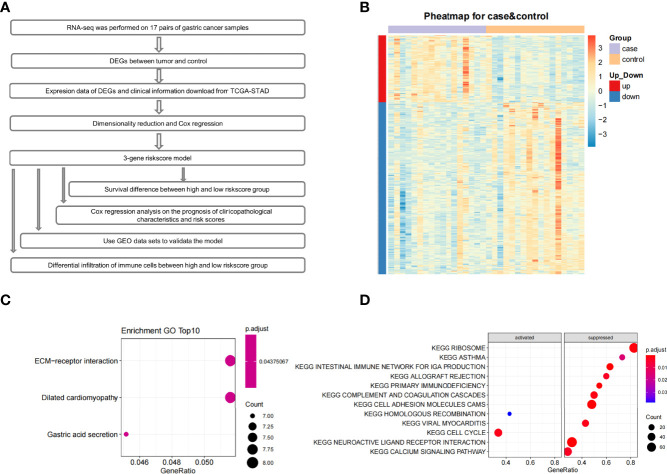
The flow chart, expression profile, GO enrichment analyses and GSEA enrichment analysis. **(A)** The flow chart of the whole article. **(B)** Heatmap depicted the expression profile of 321 significant DEGs genes between control and tumor tissues. **(C)** GO results of differentially expressed genes. **(D)** Pathway enrichment analysis of the ranked all genes by Log2FoldChange using GSEA.

The entire study design is shown as the flow chart in **Figure 1A**. Through differential expression analysis on paired tumor-normal samples from 17 GC patients, we obtained 321 DEGs **(** **Supplementary Table 3** **)**. Compared with adjacent control tissues, 90 genes were significantly up-regulated, and 231 genes were significantly down-regulated in tumors (**Figure 1B**). The GO enrichment analysis on the DEGs revealed that ‘ECM-receptor interaction’, ‘Dilated cardiomyopathy’, and ‘Gastric acid secretion’ pathways were enriched (**Figure 1C**). Among them, ECM structure is known to provide critical physical guidance during tumorigenesis, affecting cell migration, invasion, and metastasis (10, 11).

Additionally, Gene Set Enrichment Analysis (GSEA) was performed to identify gene sets or pathways in tumor and adjacent tissues. The results indicated that the ‘cell cycle-associated’ gene set was activated. Gene sets in ‘intestinal immune network for IGA production’, ‘ribosome’, ‘allograft rejection’, ‘primary immunodeficiency’, ‘complement and coagulation cascades’, ‘cell adhesion molecules cams’, ‘hematopoietic cell lineage’, ‘viral myocarditis’, ‘neuroactive ligand-receptor interaction’, and ‘calcium signaling’ pathways were in a suppressed state (**Figure 1D**).

Previous cohort studies found that patients with Immunoglobulin A (IgA) deficiency have a moderately increased risk of cancers, especially GC (12). Many molecules related to cell adhesion and glycosylation changed significantly in the tumor microenvironment. Downregulation of cell adhesion molecules would then reduce tumor cell interactions with other cells and extracellular matrix proteins (13), thereby making tumor cells more prone to migration. Finally, cell cycle pathways involved in cell growth and division were significantly activated in early GC in our study. Therefore, the results of GSEA enrichment showed that early GC may be induced by the suppression of the mucosal immune system, cell adhesion molecules, and cell cycle disorders.

### Construction and Verification of Prognostic Model

Based on the results above, the set of DEGs between GC and adjacent tissue may play a greater or lesser role in the occurrence and development of early GC, and their physiological and immunologic changes may also involve in progression of later stage tumors. It is reasonable to assume that certain DEGs have clinical prognosis significance. In order to verify this hypothesis and explore the possibility of building a prognostic evaluation system for GC, we established a prognostic model based on DEGs. The training dataset we used is TCGA-STAD (347 patients), but we only focused on the expression of 321 DEGs found in our data, since those DEGs are specifically identified during the early phase of tumors.

**Figure 2:**
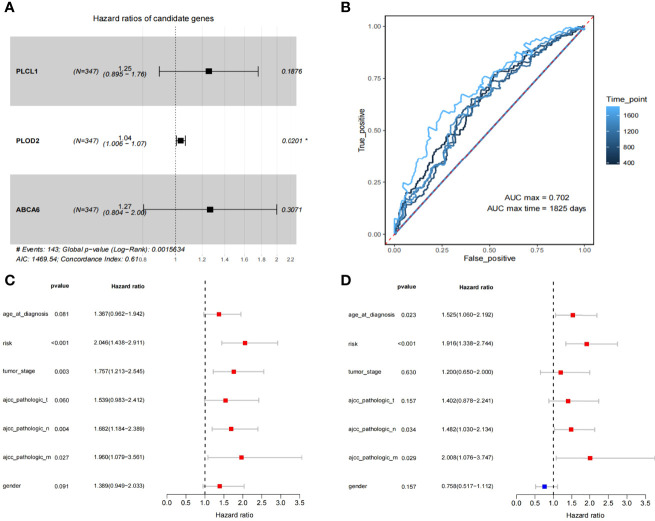
The identification of 3 prognosis related genes and construction of the 3-gene model. **(A)** Multivariate Cox regression analysis to get 3 prognosis related genes. **(B)** The ROC for survival prediction models by 1-5 years **(C, D)** The univariate and multivariate Cox regression analysis for risk-score and the prognosis of clinicopathological characteristics.

A total of 82 survival-related genes (minprop=0.45, *P*<0.05) were obtained using the best separation method in the R package ‘survminer’ for each gene in the 321 DEGs. After applying the least absolute shrinkage and selection operator (LASSO) to these 82 genes, three genes (*PLCL1*, *PLOD2*, and *ABCA6*) were finally screened. A multivariate Cox proportional hazards regression analysis on the three genes showed that the three genes met the ‘Proportional hazards Test’ (*P*=0.117, 0.993, and 0.393, respectively). The hazard ratios of three candidate genes are shown in **Figure 2A**, and the global Log-Rank p-value is 0.00156. After Cox analyses of multi-dimensional variables, the calculation formula of the overall survival risk score is established: the risk score = β1*exp1+β2*exp2+β3*exp3 (Equations 1).

We have plotted ROC curves for survival prediction model built on the 3-gene risk score, where the best cutoff value of the risk score is 0.47 (**Figure 2B**). In order to evaluate the clinical significance of risk scores, univariate and multivariate Cox regression analyses were performed to compare clinical features with risk scores (**Figures 2C, D** **)**. The results showed that ‘risk score’ (*P*<0.001) had better guiding significance than other clinical conditions, suggesting that ‘risk score’ is an independent risk factor for GC patients.

**Figure 3:**
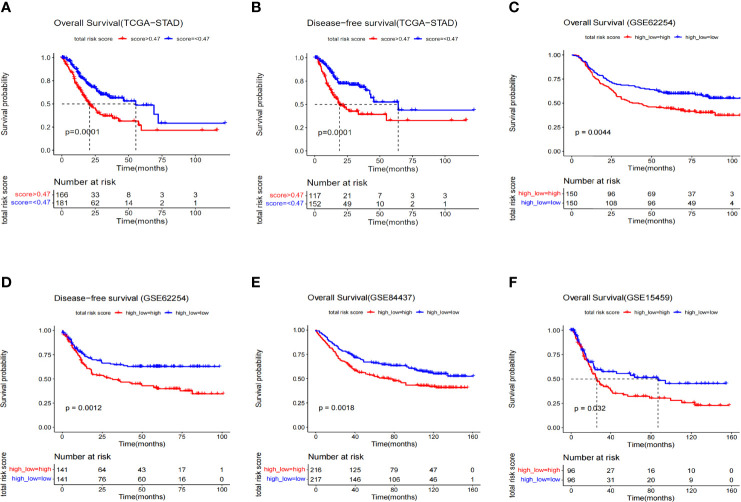
Kaplan–Meier survival analysis of different data sets composed of GC. **(A)** Overall survival curve of risk groups distinguished by cutoff value 0.47 (TCGA). **(B)** Disease-free survival curve of risk groups distinguished by cutoff of value 0.47 (TCGA). **(C)** Overall survival curve of risk groups distinguished by median value (GSE62254). **(D)** Disease-free survival curve of risk groups distinguished by median value (GSE62254). **(E)** Overall survival curve of risk groups distinguished by median value (GSE84437). **(F)** Overall survival curve of risk groups distinguished by median value (GSE15459).

Samples from TCGA training dataset were divided into two groups with significant differences in overall and disease-free survival by the 3-gene risk score (*P*=0.0001) (**Figures 3A, B** **)**. To test the robustness of the model in predicting survival in GC patients, we applied the model on three independent datasets, including GSE62254 (300 patients), GSE84437 (433 patients) and GSE15459 (192 patients) from the GEO database (**Figures 3C–F**, **Supplementary Table 4**). Since the verification data set is microarray data and it has a significant system difference compared with RNA-seq data, the risk-score cutoff value of 0.47 in this study is not applicable. Therefore, the median risk score value was applied to group separation of GEO patients. GSE84437 and GSE15459 only have overall survival data, and show the statistical difference in survival time between the high- and low- risk score groups. In addition, considering that the survival rate of gastric cancer also depends on whether the patients received neoadjuvant or adjuvant chemo/radio-treatment, in order to demonstrate the generalizability of the predictive power of the model, we also selected patients who received treatment (radiotherapy) in the TCGA database for survival analysis. The results showed that the model still had good prediction performance **(** **Supplementary Figure 1** **)**. In short, these data have proven the accuracy of our model and the importance of the three genes.

### Relation Between Immune Infiltration and Risk Score

**Figure 4:**
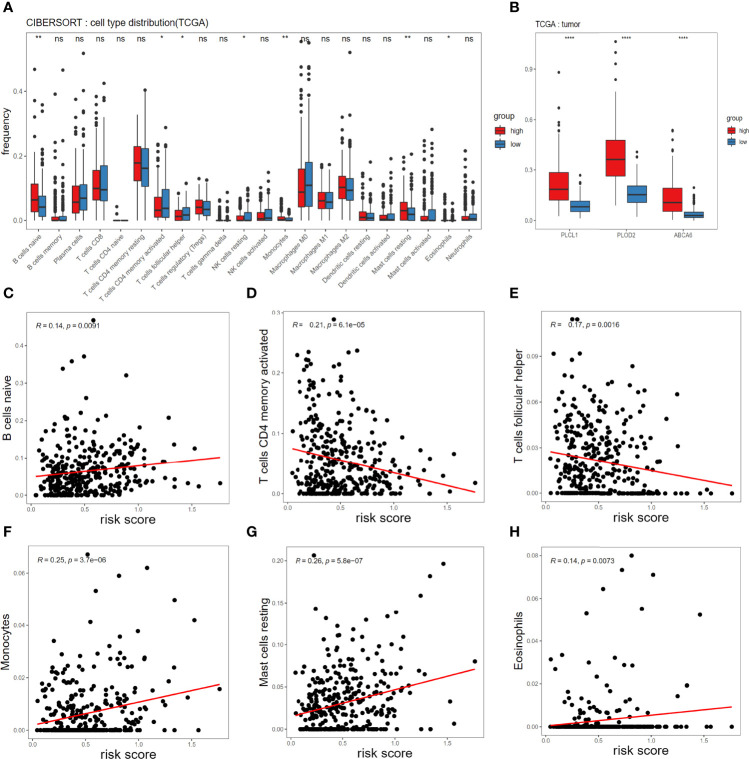
Associations of immune cell infiltration level with the risk score in TCGA. **(A)** Comparison of compositional fractions of 22 types of immune cells between the high-risk and low-risk groups evaluated using the CIBERSORT formula. **(B)** Expression comparison of PLCL1, PLOD2, ABCA6 genes between high-risk and low-risk groups. **(C-H)** Correlations between the risk model and infiltration abundances of six types of immune cells including B cell naive **(C)**, T cells CD4 memory activated **(D)**, T cells follicular helper **(E)**, Monocytes **(F)**, mast cells resting **(G)**, eosinophils **(H)**. The significance test uses t-test *p < 0.05, **p < 0.01, ***p < 0.001 and ****p < 0.0001.

To understand mechanism underlying survival-associated risk scores, we considered the infiltration of immune cells in different groups in TCGA (**Figure 4A**). In the high-risk group, gammadelta T cells, resting mast cells, M2 macrophages, resting memory CD4+ T cells, and naive B cells show high expression. While activated dendritic cells (DCs), resting NK cells, activated memory CD4+ T cells, plasma cells, and M0 macrophages show high expression in the low-risk group. In addition, the expression of PLCL1, PLOD2 and ABCA6 in the high-risk group was consistently significantly higher than that in the low-risk group, referring to the hazard ratios of the three candidate genes (**Figure 2A**), so they could be identified as high-risk genes (**Figure 4B**). Then we analyzed the correlation between 3-gene risk score and immune responses. Our risk model is positively correlated with naive B cells, Monocytes, resting mast cells and Eosinophils, but negatively associated with activated memory CD4+ T cells, T cells follicular helper and resting NK cells (**Figures 4C–H**).

**Figure 5:**
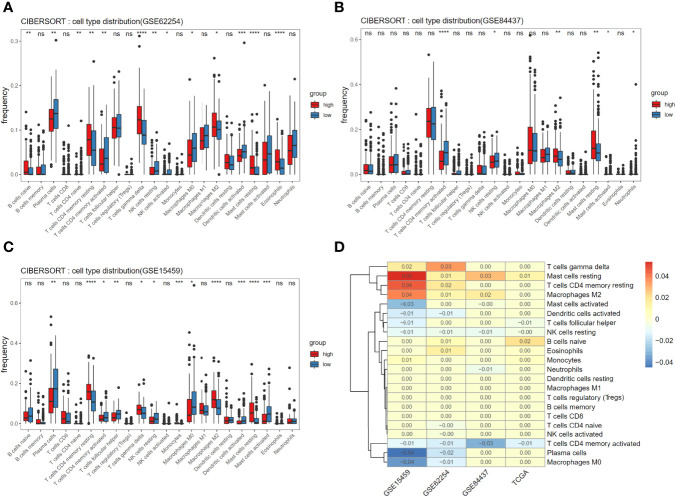
Associations of immune cell infiltration level with the risk score and consistent assessment of immune infiltration. **(A-C)** Comparison of compositional fractions of 22 types of immune cells between the high-risk and low-risk groups in GSE62254 **(A)**, GSE84437 **(B)**, GSE15459 **(C)**. **(D)** The significant difference of cells proportion between groups was presented in the form of heat map. Non-zero value means that there is significant difference in t-test results between high and low score groups and the value is the mean difference of cell component proportion between high group and low group. *p < 0.05, **p < 0.01, ***p < 0.001 and ****p < 0.0001.

In addition to studying the immune infiltration in the TCGA training set, we also observed immune infiltration in the GEO validation set **(** **Figures 5A–C** **)**, and finally focused on immune cells with significant and consistent changes in at least 2 datasets **(** **Figure 5D** **)**.

New evidence suggests that resident memory T cells (TRM) play a special role in solid GC tumors. The appearance of TRM cells in solid tumors is associated with cancer progression (14). DC-originated cognate memory CD4+ T cells can influence the expansion, transportation, and differentiation of secondary CD8+ T cells, thereby significantly increasing control on tumor growth (15). The DCs are a diverse group of special antigen-presenting cells essential for initiating and regulating innate and adaptive immune responses. Therefore, the activation of DC is also reasonable for tumor immune enhancement (16). After mast cells are activated, other cells such as effector and regulatory immune cells and DCs can be recruited to respond (17). On the other hand, resting mast cells fail to play an active immune role in recruitment. Consistent with our results, plasma cell content is shown as proportional to survival of triple-negative breast cancer and adenocarcinoma of the esophagogastric junction cancer (18, 19). M2 macrophages are associated with poor prognosis and proliferation in many tumors (20). By combining the infiltration of immune cells, we speculate that there is a favorable microenvironment for tumor growth in the high-risk group. The microenvironment of high-risk group contains the low expression of activated memory CD4+ T cells, activated DCs and plasma cells, and high expression of M2macrophage, resting memory CD4+ T cells, and resting mast cells, which leads to a poor clinical prognosis. These speculations are consistent with our correlation analysis. Our higher risk score is related to mast cells that failed to function and B cells that are immature, while when T cells are activated, our risk score will decrease significantly, reflecting the strong links between immune cells and risk model.

## Discussion

The GC ranks fifth in incidence and third in mortality (21). Like other malignant tumors, the best treatment method is to perform tumor removal surgery after early diagnosis, preventing the spread of tumor cells (22). However, other treatments are needed for patient with advanced GC or patient whose physical condition is not suitable for surgery. Although chemotherapy and radiotherapy are more effective for those GC patients, their side effects are severe (23). Molecular targeted therapy has been a research hot spot in recent years, especially for advanced GC. These selected tumor markers are useful for treating aggressive and metastatic tumors. For example, the high expression of YAP1 in advanced gastric adenocarcinoma endows tumors with strong invasion and metastasis ability, thus leading to the occurrence of peritoneal cancer in 45% of gastric adenocarcinoma patients (24). Therefore, inhibiting the expression of YAP1 can effectively inhibit tumor migration and achieve the purpose of treatment (24). However, few genes have been identified as effective targets for treating GC. There are mainly five categories of molecular targeted drugs applicable for GC, which are targeting human epidermal growth factor receptor 2 (HER2), epidermal growth factor receptor (EGFR), vascular endothelial growth factor (VEGF), Claudin 18.2 (CLDN 18.2) and mammalian target of rapamycin (mTOR) (25). Continuous research is needed to bring more suitable treatment options to patients. In this study, 321 DEGs were identified between tumor and adjacent area tissue, and a risk model of prognostic-related genes was constructed. Validation of multiple data sets shows that *ABCA6*, *PLCL1*, and *PLOD2* can jointly predict disease-free survival and overall survival and may serve as potential targets for treating GC, providing a basis for developing targeted drugs.

Cancer-related genes are vital for the development of tumors (26). The protein encoded by *PLOD2* mediates the hydroxylation of lysine residues of collagen, thereby mediating cross-linking between collagens. PLOD2 plays a role in various cancers, such as renal clear cell carcinoma, laryngeal cancer, esophageal squamous cell carcinoma, breast cancer, osteosarcoma, liver cancer, and lung cancer (27). The overexpression of *PLOD2* is related to the occurrence of epithelial-to-mesenchymal (EMT), which is one of the main steps leading to metastasis of tumors (28). In the study of glioma, PLOD2 is induced by hypoxia-inducible factor-1α (HIF-1α) and then activates the PI3K/AKT signaling pathway, which eventually leads to the occurrence of EMT (29). MiR-26a-5p and miR-26b-5p can regulate PLOD2, and PLOD2 is a potential prognostic marker for bladder cancer (30). PLOD2 can up-regulate BCRP, thus promoting the resistance of GC cells to 5-fluorouracil (31). PLOD2, a new regulator of glucose metabolism, plays a role in controlling the expression of HK2 in CRC cells, indicating that PLOD2 is a therapeutic target and inhibition of which can benefit patients (32). PLCL1 is a phospholipase that participates in calcium ion binding and proton pump-related pathways. Its abnormal expression may contribute to the occurrence and development of cancer. *PLCL1*, as a survival-related gene of GC, is also significantly correlated with clinical characteristics, tumor microenvironment immune cells, tumor mutation burden (TMB), and tumor necrosis factor (TNF) (33). *ABCA6* is related to the survival time of patients with hepatocellular carcinoma, but its relationship with the prognosis of GC is unclear (34). In short, existing research results are consistent with our findings that three genes are associated with poorer prognosis, and our research has provided new insights on basis for future GC research.

In addition to the association with disease development described above, cbioportal co-expression analysis in our study also showed that these three genes were positively correlated with the expression of LYAN and negatively correlated with the expression of CDH1(**Supplementary Figure 2**), the two well-known genetic marker genes associated with gastric cancer. The high expression of LAYN was significantly associated with poor overall survival (OS) and progression-free survival (PFS) in gastric cancer patients (35). Moreover, LAYN expression is correlated with diverse immune infiltration levels in cancer, especially in gastric cancer, there is a moderate to strong positive relationships between LAYN expression level and infiltration level of monocyte and M2 macrophages (35), which consistent with our immune assessment results, the high-risk group had higher infiltration levels of monocytes and M2 macrophages compared to the low-risk group. *CDH1* is the main gene involved in hereditary GC, encoding the E-cadherin protein, whose germline mutations are responsible for Hereditary Diffuse Gastric Cancer (HDGC) (36). Furthermore, abnormal E-cadherin expression was significantly associated with RFS and overall survival OS (*p* = 0.003 and *p* = 0.001, respectively) (37). Therefore, there may be a regulatory network between these genes to jointly regulate the occurrence and progression of the disease.

In terms of the immune microenvironment, there are prominent differences in some immune cells between the high-risk and low-risk groups. In the high-risk group, the expressions of M2 macrophages, resting mast cells, resting memory CD4+ T cells, and naive B cells are high, while the expressions of activated memory CD4+T cells, activated DCs, plasma cells, and M0 macrophages are low. Mast cells and DCs are the first groups of cells in the immune system to interact with allergens, other antigens, and invading pathogens in the environment (38,39). When both types of cells are in resting states, their functions cannot be exercised, and tumor immune escape may occur. The tight correlation between our model and immunity suggests that our model perfectly reflects the immune status of the predicted samples.

A 3-gene prognosis model related to early GC was established in our study. Patients were stratified according to risk levels. Patients with high-risk scores had significantly lower overall prognostic and disease-free survival than patients with low-risk scores. Therefore, the three genes may be used as clinical biomarkers to monitor the biological changes in early tumor development, rather than taking measures until the tumor progresses to a certain extent. We may also take necessary measures to prevent recurrence or develop targeted drugs for early tumor events by monitoring the changes in this signature. Our study also has some limitations. The sample size for discovery of DEGs is limited, and the research is only from the perspective of bioinformatics. Thus, more *in vitro* and *in vivo* experiments are needed to confirm these conclusions. In the future, our laboratory will verify the specific roles of these three prognosis-related genes in the efficacy of immunotherapy in gastric cancer patients.

## Data Availability Statement

The data presented in the study are deposited in the CNGB repository, accession number CNP0002454.

## Ethics Statement

The studies involving human participants were reviewed and approved by the institutional review board of BGI. The patients/participants provided their written informed consent to participate in this study. Written informed consent was obtained from the individual(s) for the publication of any potentially identifiable images or data included in this article.

## Author Contributions

SX, TZ, JY, and GL conceived the project and provided analytical ideas. SD conducted experimental operations. SX, TZ, and JY performed the data analysis, plotted the figures, and wrote the manuscript. GL, JM, XZ, TL, and CW reviewed the manuscript carefully and made suggestions. CL revised the manuscript and provided suggestions to data analysis. All authors contributed to the article and approved the submitted version.

## Funding

This work was supported by China National GeneBank (CNGB). Science, Technology and Innovation Commission of Shenzhen Municipality under grant (No.JCYJ20170412153155228).

## Conflict of Interest

The authors declare that the research was conducted in the absence of any commercial or financial relationships that could be construed as a potential conflict of interest.

## Publisher’s Note

All claims expressed in this article are solely those of the authors and do not necessarily represent those of their affiliated organizations, or those of the publisher, the editors and the reviewers. Any product that may be evaluated in this article, or claim that may be made by its manufacturer, is not guaranteed or endorsed by the publisher.
