# Supplementary material for: Identification of a 3-Gene Model as Prognostic Biomarker in Patients With Gastric Cancer
Source: Front Oncol. 2022 Jul 14;12:930586. doi: 10.3389/fonc.2022.930586 (PMC9329618; doi:10.3389/fonc.2022.930586)

# Supplementary Figure 1

To verify the reliability of our model predictions, we performed a survival analysis on 71 patients who treated with radiotherapy at TCGA. The analysis results show that our model still shows a good prediction effect.

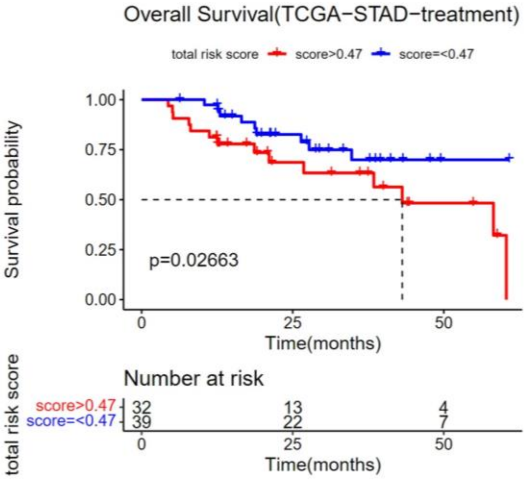

## Supplementary Figure 2

*LAYN* and *CDH1* are two well-known gastric cancer genetic markers that we chose to investigate the correlation with our 3-gene signature, for the reasons as following:

Cbioportal co-expression analysis showed that these three genes were positively correlated with the expression of *LYAN* and negatively correlated with the expression of *CDH1*. The dataset type used was gastric adenocarcinoma (TCGA, Firehose Legacy) (441 patients/sample)

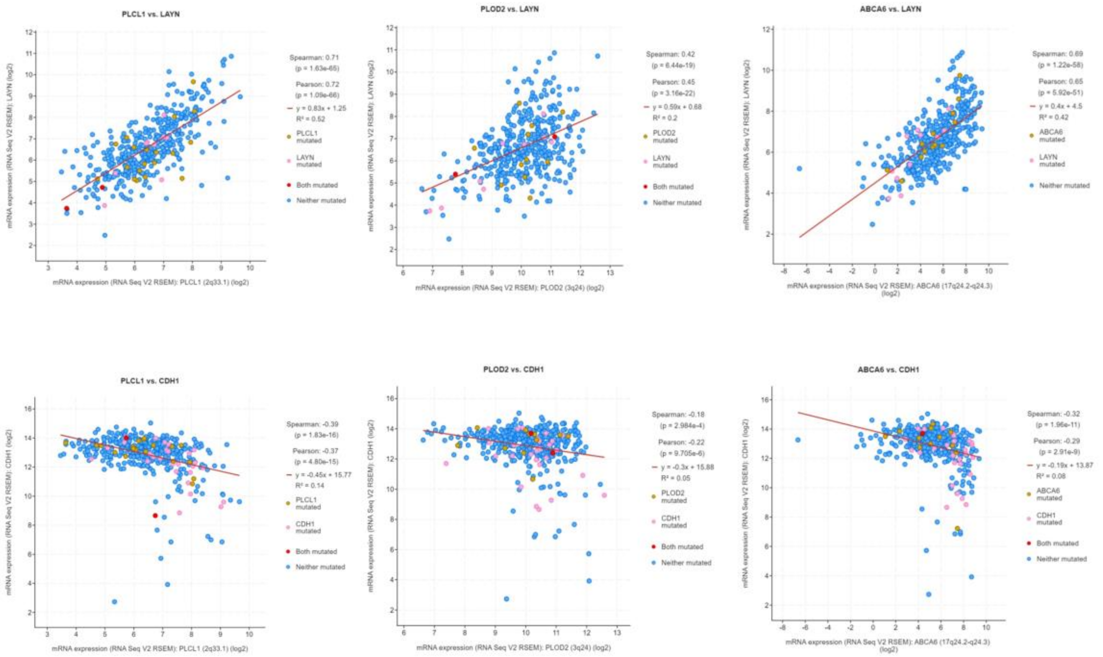

Supplement: Supplementary file 1 [file DataSheet_1.pdf]
